# A Meta-Analysis of Core Stability Exercise versus General Exercise for Chronic Low Back Pain

**DOI:** 10.1371/journal.pone.0052082

**Published:** 2012-12-17

**Authors:** Xue-Qiang Wang, Jie-Jiao Zheng, Zhuo-Wei Yu, Xia Bi, Shu-Jie Lou, Jing Liu, Bin Cai, Ying-Hui Hua, Mark Wu, Mao-Ling Wei, Hai-Min Shen, Yi Chen, Yu-Jian Pan, Guo-Hui Xu, Pei-Jie Chen

**Affiliations:** 1 Department of Sport Rehabilitation, Shanghai University of Sport, Shanghai, China; 2 Department of Rehabilitation Medicine, Huadong Hospital Affiliated to Fudan University, Shanghai, China; 3 Department of Rehabilitation Medicine, Shanghai Gongli Hospital, Shanghai, China; 4 Department of Exercise and Sport Science, Shanghai University of Sport, Shanghai, China; 5 Department of Orthopaedics and Rehabilitation, Ninth People’s Hospital Affiliated to Shanghai Jiaotong University Medical School, Shanghai, China; 6 Department of Sport Medicine, Huashan Hospital Affiliated to Fudan University, Shanghai, China; 7 Department of Rehabilitation and Ancillary Services, Gleneagles International Medical and Surgical Center, Shanghai, China; 8 Chinese Evidence-based Medicine, West China Hospital of Sichuan University, Chengdu, China; 9 Department of Orthopaedics and Trauma Surgery, Huadong Hospital Affiliated to Fudan University, Shanghai, China; The James Cook University Hospital, United Kingdom

## Abstract

**Objective:**

To review the effects of core stability exercise or general exercise for patients with chronic low back pain (LBP).

**Summary of Background Data:**

Exercise therapy appears to be effective at decreasing pain and improving function for patients with chronic LBP in practice guidelines. Core stability exercise is becoming increasingly popular for LBP. However, it is currently unknown whether core stability exercise produces more beneficial effects than general exercise in patients with chronic LBP.

**Methods:**

Published articles from 1970 to October 2011 were identified using electronic searches. For this meta-analysis, two reviewers independently selected relevant randomized controlled trials (RCTs) investigating core stability exercise versus general exercise for the treatment of patients with chronic LBP. Data were extracted independently by the same two individuals who selected the studies.

**Results:**

From the 28 potentially relevant trials, a total of 5 trials involving 414 participants were included in the current analysis. The pooling revealed that core stability exercise was better than general exercise for reducing pain [mean difference (−1.29); 95% confidence interval (−2.47, −0.11); P = 0.003] and disability [mean difference (−7.14); 95% confidence interval (−11.64, −2.65); P = 0.002] at the time of the short-term follow-up. However, no significant differences were observed between core stability exercise and general exercise in reducing pain at 6 months [mean difference (−0.50); 95% confidence interval (−1.36, 0.36); P = 0.26] and 12 months [mean difference (−0.32); 95% confidence interval (−0.87, 0.23); P = 0.25].

**Conclusions:**

Compared to general exercise, core stability exercise is more effective in decreasing pain and may improve physical function in patients with chronic LBP in the short term. However, no significant long-term differences in pain severity were observed between patients who engaged in core stability exercise versus those who engaged in general exercise.

**Systematic Review Registration:**

http://www.crd.york.ac.uk/PROSPERO PROSPERO registration number: CRD42011001717.

## Introduction

Low back pain (LBP) is one of the two most common types of disability affecting individuals in Western countries (the other is mental illness), and the assessment of LBP-related disabilities represents a significant challenge [1]. LBP affects approximately 80% of people at some stage in their lives [2], [3]. In developing countries, the 1-year prevalence of LBP among farmers was 72% in southwest Nigeria [4], 56% in Thailand [5], and 64% in China [6]. The impact of chronic LBP can be severe and profound because chronic LBP often results in lost wages and additional medical expenses and can even increase the risk of incurring other medical conditions [7], [8]. In the United States, the total indirect and direct costs due to LBP are estimated to be greater than $100 billion annually [9], [10].

Exercise therapy seems to be an effective treatment to relieve the pain and to improve the functional status of patients with chronic LBP in most clinical practice guidelines [11]. Core stability training has become a popular fitness trend that has begun to be applied in rehabilitation programs and in sports medicine [12]. Many studies [13]–[15] have shown that core stability exercise is an important component of rehabilitation for LBP. Panjabi [16] proposed a well-known model of the spine stability system that consists of three subsystems: the passive subsystem (which includes bone, ligament and joint capsule), the active subsystem (which includes muscle and tendons), and the neural subsystem (which consists of the central nervous system and peripheral nervous system). According to this model, these three subsystems work together to provide stabilization by controlling spinal movement. Thus, an effective core stability exercise should consider the motor and sensory components of the exercise and how they relate to these systems to promote optimal spinal stability [17]. In addition, core stability training includes the exercise associated with the prior activation of the local trunk muscles and should be advanced to include more intricate static, dynamic, and functional exercises that involve the coordinated contraction of local and superficial spinal muscles.

**Figure 1:**
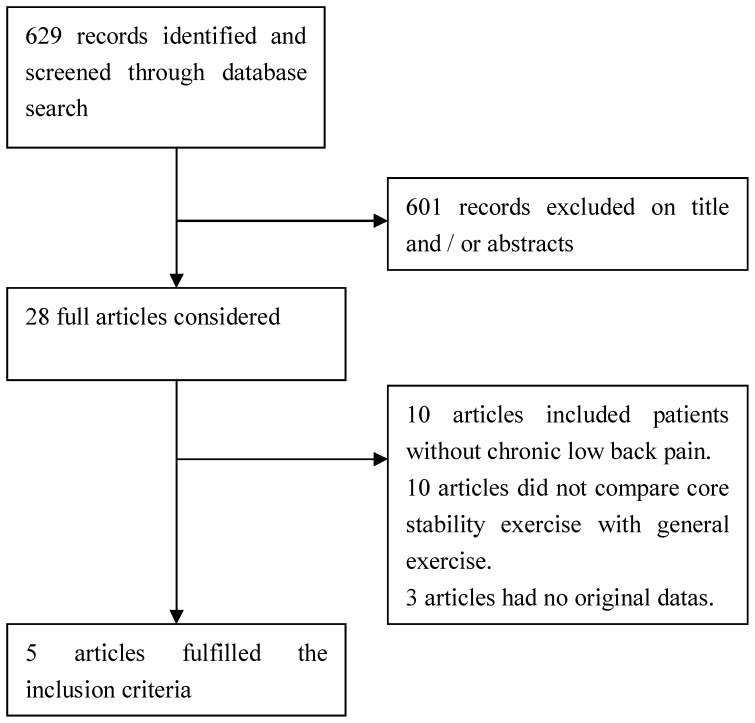
Flow chart of the study selection procedure.

**Table 1:** Characteristics of Included Studies.

Article	Patient Characteristic, Sample Size, andDuration of Complaint,year	Core stabilizationexercise group	General exercise group	Outcomes	Follow up
Manuela 2007(Brazil)	aged 18–80;n = 240;Duration ofLBP>3mon;	n = 80(age: 51.9±15.3); retrainingspecific trunk muscles usingultrasound feedback; 12 treatmentsessions over 8 weeks	n = 80(age: 54.8±15.3);strengthening, stretching and aerobic exercises; 12 treatment sessions over 8 weeks	pain(VAS) and disability (Roland Morris Disability Questionnaire)	8 weeks 6month 12month
Monica 2010(Norway)	aged 19–60;n = 72; Duration of LBP>3mon;	n = 36(age:40.9±11.5); motorcontrol exercise; once a week for8 weeks;	n = 36(age:36.0±10.3); trunk strengthening and stretching exercises; once a week for 8 weeks	pain(NRS 0–10)disability (ODI)	8 weeks 12month
Fabio 2010(Brazil)	n = 30; Duration of LBP>3 month;	n = 15(age: 42.07±8.15); segmentalstabilization exercises; twice perweek for 6 weeks	n = 15(age: 41.73±6.42); superficial strengthening exercise(n = 15); twice per week for 6 weeks	pain(VAS, McGill) and disability (ODI)	6 weeks
Ottar 2010(Norway)	aged 19–60;n = 72;Duration ofLBP>3mon;	n = 36(age:43.4±10.2); slingexercise; once a week for8 weeks	n = 36(age:36.0±10.3); trunk strengthening and stretching exercises; once a week for 8 weeks	pain(NRS 0–10)disability (ODI)	8 weeks 12month
Padmini 2008(India)	aged 18–60;n = 80;Duration ofLBP>3mon;	n = 40(age:49±3.6); traditionalyoga scriptures;1 week	n = 40(age:48±4); physical exercises(n = 40); 1 week	disability (ODI)	1 weeks

Abbreviations: LBP, low back pain; ODI, Oswestry Disability Index; VAS, Visual Analog Scale; NRS, Numerical Rating Scale.

Although there have been four published systematic reviews [18]–[21] of core stability training, these articles only include a review of the literature published prior to June 2008. Positive effects have been reported with different forms of exercise used by physical therapists. However, it is currently unclear whether core stability training produces more beneficial effects than conventional exercise for patients with chronic LBP.

Core stability training has a powerful theoretical foundation for the prevention and treatment of LBP, as is evidenced by its widespread clinical use. However, there appears to be no consensus agreement that core stability exercise is better than general exercise for chronic LBP. It is important to ensure that the determination of the most effective exercise for LBP is based on scientific evidence so as not to waste staff time and resources and to avoid unnecessary stress for patients with LBP and their families. The purpose of this paper is to conduct a meta-analysis of the effects of core stability exercise compared to general exercise as a treatment for chronic LBP.

**Table 2:** Risk of Bias Assessment of Included Studies.

Article	Random sequence generation	Allocation concealment	Blinding of participants and personnel	Blinding of outcome assessment	Incomplete outcome data	Selective reporting	Other bias	Risk of bias
Manuela 2007(Brazil)	low	low	low	low	low	low	high	high
Monica 2010 (Norway)	low	low	low	low	low	low	high	high
Fabio 2010 (Brazil)	low	low	unclear	low	low	low	high	high
Ottar 2010 (Norway)	low	unclear	low	low	low	low	high	high
Padmini 2008 (India)	low	low	high	low	low	low	high	high

**Figure 2:**
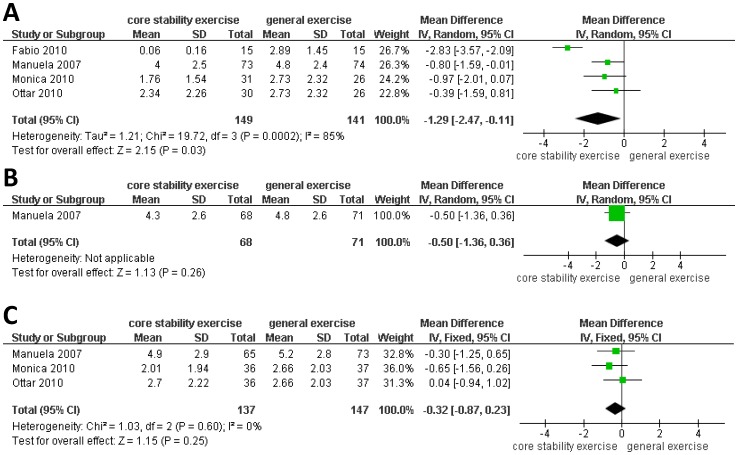
Meta-analyses of core stability exercise versus general exercise effect on pain. A: mean difference (MD) at the end of the intervention (not longer than 3 months). B: MD at six months. C: MD at long-term follow-up (12 months or more).

**Figure 3:**
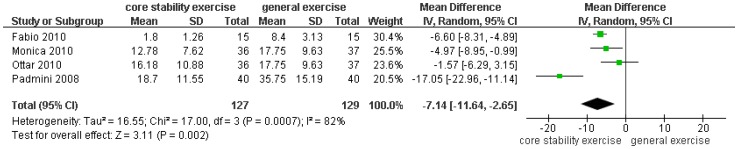
Meta-analyses of core stability exercise versus general exercise effect on back-specific functional status (Oswestry Disability Index, ODI): mean difference (MD) at the end of the intervention (not longer than 3 months).

## Methods

### Search Strategy

We identified randomized controlled trials (RCTs) by electronically searching the following databases: China Biology Medicine disc (1970–October 2011), PubMed (1970–October 2011), Embase (1970–October 2011), and the Cochrane Library (1970–October 2011).

A detailed explanation of the full electronic search strategy for PubMed is presented in Appendix S1. Briefly, the following medical subject headings (MeSH) were included: low back pain, sciatica, lumbosacral region, exercise, and chronic pain. The keywords used were RCTs, double-blind method, single-blind method, random allocation, pelvic girdle pain, motor control, exercise therapy, stability, stabilization, general exercise, traditional exercise, conventional exercise, specific exercise, and physical therapy. We removed duplicates that were identified in multiple database searches.

### Inclusion Criteria

#### 1. Types of studies

Only RCTs examining the effects of core stability exercise versus general exercise for the treatment of patients with chronic LBP were included. No language or publication date limits were set.

#### 2. Types of participants

We included articles with both female and male subjects (over 18 years of age) who had chronic LBP (longer than 3 months). We excluded articles that included participants with LBP evoked by specific conditions or pathologies.

#### 3. Types of interventions

We included articles that compared a control group, which received general exercise, and a treatment group, which received core stability exercise training. A core stability training program could be described as the reinforcement of the ability to insure stability of the neutral spine position [22]. Core stability exercises were usually performed on labile devices, such as an air-filled disc, a low density mat, a wobble board, or a Swiss ball [23].

#### 4. Types of outcome measures

The primary outcomes of interest were pain intensity, back-specific functional status, quality of life, and work absenteeism. Outcomes were recorded for three time periods [11]: long term (1 year or more), intermediate (6 months), and short term (less than 3 months).

### Selection of Studies

Two reviewers (Wang XQ, Bi X) used the pre-specified criteria to screen for relevant titles, abstracts and full papers. An article was removed if it was determined not to meet the inclusion criteria. If these two reviewers reached different final selection decisions, a third reviewer (Zheng JJ) was consulted.

### Data Extraction

We extracted the following data from the included articles: study design, subject information, description of interventions between the control and experimental group, follow-up period, and outcome measures. These data were then compiled into a standard table. The two reviewers who selected the appropriate studies also extracted the data and evaluated the risk of bias. It was necessary to consult an arbiter (Zheng JJ) to reconcile any disagreements.

### Assessing the Risk of Bias

We used the Cochrane Collaboration recommendations [24] to assess the risk of bias for all articles. The following information was evaluated: random sequence generation, allocation concealment, blinding of participants and personnel, blinding of outcome assessments, incomplete outcome data, selective reporting and other bias. Two reviewers (Xu GH, Hua YH) evaluated the methodological quality of all articles examined in the current study. An arbiter was consulted (Chen PJ) to reconcile any disagreements.

### Statistical Analysis

Review Manager Software (RevMan5.2) was used for the meta-analysis. Heterogeneity among the studies was evaluated using the I^2^ statistic and the chi-squared test. The fixed effects model was used if the heterogeneity test did not reveal statistical significance (I^2^<50%; P>0.1). Otherwise, we adopted the random effects model. We conducted a sensitivity analysis if heterogeneity existed among the studies. All of the variables in the studies included in this meta-analysis were continuous, so we used the mean difference (MD) and 95% confidence interval (CI) to analyze the studies. We considered P values less than 0.05 to be statistically significant.

Systematic review registration: http://www.crd.york.ac.uk/PROSPERO. PROSPERO registration number: CRD42011001717.

## Results

### Search Results

The process of identifying eligible studies was outlined in figure 1. Six hundred twenty-nine records were initially identified through the Cochrane Library, PubMed, Embase, and China Biology Medicine disc. Of these, 28 potentially eligible articles were included based on their title and abstract. After reviewing these 28 potential articles, only 5 articles [25]–[29] fulfilled the inclusion criteria. The remaining 23 articles [30]–[52] were removed because the trials included participants with diagnoses other than chronic LBP, did not compare core stability exercise with general exercise, or the original data were not available from the authors. The characteristics of each included study are described in table 1.

### Risk of Bias of Included Studies

According to the Cochrane Collaboration recommendations, each article was at a high risk of bias. Thus, the evidence involved in this meta-analysis had a high overall risk of bias. Each article was described as randomized, but the randomization method was unclear for one study [27]. Four articles used the allocation concealment method, but we could not determine the allocation concealment in the Ottar 2010 article [28]. Three of the included articles attempted to blind the participants to the allocated treatment, and outcome assessors were blinded in five trials. Incomplete outcome data were at a low risk of bias in all articles. The risk of bias assessment of all included studies is described in table 2.

### Core Stability Exercise Versus General Exercise on Pain Intensity

In total, four trials assessed pain intensity using a numeric rating scale (NRS) and a visual analog scale (VAS). The data indicated that core stability exercise was better than general exercise for short-term pain relief when the results were combined in a random-effects model [MD (95% CI) = −1.29 (−2.47, −0.11), P = 0.003] [Fig. 2(A)]. However, no significant differences were observed between the effects of core stability exercise and general exercise at 6 months [MD (95% CI) = −0.50 (−1.36, 0.36), P = 0.26] [Fig. 2(B)] or 12 months [MD (95% CI) = −0.32 (−0.87, 0.23), P = 0.25] [Fig. 2(C)].

### Core Stability Exercise Versus General Exercise on Disability

Five studies included self-reported back-specific functional status. Of these, one used the Roland Morris Disability Questionnaire (RMDQ), and four used the Oswestry Disability Index (ODI). Compared to general exercise, core stability exercise resulted in a significant improvement in functional status by the random-effects model in the short term [MD (95% CI) = −7.14 (−11.64, −2.65), P = 0.002] (Fig. 3).

## Discussion

This meta-analysis, which included 414 patients, identified 5 RCTs that compared core stability exercise and general exercise for chronic LBP. The risk of bias was assessed for each article using the Cochrane Collaboration recommendations. In addition, each article contained a high risk of other bias. And it was difficult to evaluate whether the articles described the outcome measures they had originally meant to describe. However, no serious complications were reported in any of the five articles that investigated adverse events. However, the number of included subjects was too small to determine the safety of core stability exercise.

The results of this meta-analysis indicate that core stability exercise is better than general exercise for pain relief and improving back-specific functional status in the short term. However, no significant differences in pain relief were observed in the intermediate- and long-term follow-up periods. The primary results of this review are consistent with the findings of a systematic review [20] of the effects of core stability exercise on nonspecific LBP. The results of the meta-analysis indicated that core stability exercise can be more effective than other types of exercise in improving back-specific functional status in the short term (MD = −5.1points, 95% CI = −8.7 to 1.4). Two other systematic reviews [18], [19] also reported that specific stabilization exercise was better than ordinary medical care and treatment by a general practitioner for reducing pain over the short term and intermediate term.

Compared to the prior reviews, approximately four-fifths of the articles included in the current study were new, and all of the articles in the current analysis considered only patients with chronic LBP (duration of pain >12 weeks). In addition, we conducted a meta-analysis of the effects of core stability exercise compared to general exercise. Because of these characteristics, this meta-analysis is considered to be much more robust.

Core stability is the ability to control the position and movement of the central portion of the body [53]. Popular fitness programs, such as Tai Chi, Yoga, and Pilates, are based on core stability exercise principles. There are several different approaches currently in use for core stability exercise for LBP, which could lead to different results. A systematic review and meta-analysis of different core stability exercises for LBP should be conducted to determine the optimal treatment approach.

### Limitations

This meta-analysis was characterized by several limitations that should be noted. The first limitation, which is common in many systematic reviews, was that the findings were based on relatively low quality data that had a high risk of bias. Although several of the articles involved in this meta-analysis were published within the last five years, methodologically rigorous articles were still deficient. Second, the total number of subjects involved in the meta-analysis was too small to identify relatively small disparities between the effects of core stability exercise and general exercise. A third limitation was that numerous articles did not contain sufficient information for evaluating the quality and clinical relevance of the data. Another limitation was the probability of publication bias, which we attempted to diminish via a substantial database search. However, unpublished articles were not searched. Finally, it would have been preferable to conduct multiple outcome measures between the compared treatments in this meta-analysis. However, the primary outcome measures evaluated in the majority of articles were pain intensity and back-specific functional status. Relatively few articles had a significant analysis of quality of life, global improvement, and return to work/absenteeism.

### Implications for Practice

In comparison to general exercise, core stability exercise may be more effective in relieving pain and improving back-specific function for patients with chronic LBP in the short term. However, no significant differences were observed between core stability exercise and general exercise in pain and functional status in the long term. However, these conclusions are sustained by low-quality data, and more definitive articles are required to confirm these results.

### Implications for Research

Articles that were methodologically sound and sufficiently powered are required to confirm the effects of core stability exercise on pain relief and functional improvements in patients with chronic LBP. The types of outcomes in articles should include proprioception, muscle strength and trunk endurance to provide insight into the potential mechanisms of cooperative action. Comparisons of different core stability exercises would be more reasonable. The effects of core stability exercise should be evaluated over the long term. Eventually, theories regarding the mechanisms by which core stability exercise relieves pain in patients with LBP should be explored further.

## Supporting Information

Appendix S1 MEDLINE search strategy.
(DOC)

Checklist S1 PRISMA 2009 Checklist
(DOC)
