# Supplementary material for: A Meta-Analysis of Core Stability Exercise versus General Exercise for Chronic Low Back Pain
Source: PLoS One. 2012 Dec 17;7(12):e52082. doi: 10.1371/journal.pone.0052082 (PMC3524111; doi:10.1371/journal.pone.0052082)
Supplement: Appendix S1 — MEDLINE search strategy. (DOC) [file pone.0052082.s001.doc]

**Appendix A. MEDLINE search strategy**

1. randomized controlled trial. pt
2. Random Allocation/
3. Randomized Controlled Trials/
4. Double-Blind Method/
5. Single-Blind Method/
6. or/ 1-5
7. Animal/ not Human
8. 6 not 7
9. low back pain. mh
10. sciatica. mh
11. lumbosacral region. mh
12. lumbago.tw
13. pelvic girdle pain. tw
14. spondylosis.tw
15. or/ 9-14
16. 8 and 15
17. motor control.tw
18. exercise. mh
19. exercise therapy.tw
20. stability.tw
21. stabilization.tw
22. stabilizing.tw
23. general exercise.tw
24. traditional exercise.tw
25. conventional exercise.tw
26. specific exercise.tw
27. pilates.tw
28. yoga.tw
29. tai chi.tw
30. physical therapy.tw
31. or/17-30
32. 16 and 31
